# Supplementary material for: Voluntary wheel running has no impact on brain and liver mitochondrial DNA copy number or mutation measures in the PolG mouse model of aging
Source: PLoS One. 2020 Mar 2;15(3):e0226860. doi: 10.1371/journal.pone.0226860 (PMC7051064; doi:10.1371/journal.pone.0226860)
Supplement: Attachment — Submitted filename: Plos_One_reviewer_Response_final_012920.docx [file pone.0226860.s002.docx]

Thank you for organizing the review process. Below the comments from the **reviewer are in bold** and our response is in plain text.

**Reviewer #1: This is a well-designed study to assess whether exercise can counteract the effects of increased mtDNA mutations induced by PolG mutation. The design and methods are thoroughly described, and this reviewer is particularly pleased with the amount of detail included in the statistical analyses. Though the majority of the data presented are 'negative', the robust experimental design and inclusion of multiple controls speaks to the reliability of the data presented. Further, these data answer the question the authors posed, and is of value to the research community.**

**I have no major concerns with this manuscript but recommend addressing these minor points in a revision:

-	…did the authors consider assessing corticosterone levels in the young vs old animals? As the authors are investigating effects of exercise in the brain, they implemented voluntary wheel-running to minimize stress, which necessitated animals being housed alone. Mice are social mammals and there is evidence of increased corticosterone in single-housed rodents. This stress response may also be age-dependent and worth measuring if plasma/serum samples are available.**

We very much appreciate the reviewer’s affirmation of our attempts to produce a study with careful design, statistical power, and methodology despite the ultimately negative results. We also, of course, hope these results are useful to scientists interested in exercise and/or mitochondria.

Unfortunately plasma samples are not available to test for corticosterone levels, but probably would have been a good idea in retrospect. The effects of stress are a particularly interesting parallel pathway in the exercise literature. As the reviewer points out, many exercise studies, including this one, probably do not sufficiently address the potential effects of stress responses, both in relation to cortisol/corticosterone and in other pathways. Indeed, the effects of social isolation are likely a confounder in many studies, not just exercise studies. Thus, we added a paragraph to the discussion (second paragraph) to address the concerns about single housing and stress.

The measuring of corticosterone levels during or across exercise interventions would likely provide a useful covariate to explore. For instance, it may be paradoxically possible that the positive effects of exercise seen in the forced treadmill running vs our free-wheel running may, in fact, be due to the severe stress and not to the exercise itself.

For consistency, sedentary animals were also housed individually, so any stress caused by social isolation was also experienced by the sedentary animals. Overall, we felt that the stress caused by forced exercise would have caused an unequal and severe amount of stress for the exercised group. Anecdotally, there is also evidence that male mice specifically experience less stress when housed individually (perhaps due to fighting) and all of the mice in the current study were male. Our rodent facility also only allows direct littermates of male mice to be co-housed due to fighting concerns which can result in forced euthanasia by veterinarian order (statistically risky). Since we split littermates deliberately into the 2 groups to attempt to control for litter effects, co-housing littermates to prevent social isolation would not be simultaneously possible.

**-	I strongly recommend a thorough copy-edit of the manuscript by the senior author to ensure consistency in acronyms, hyphens, and general writing style. The English is technically correct, but could be edited for style.**

We have done our best to edit the manuscript for consistency and have, hopefully, improved the general style.

The question of hyphenation, particularly of “wild-type” vs “wild type” caused us some reflection, as the first is appropriate to an adjective (whether the corresponding noun is present or implied), the second to a noun. We have used the un-hyphenated form only in the case where “wild type” is the name of a category in statistical analysis, that is, a group name.
